# Parental Health and Flourishing Among Young US Children

**DOI:** 10.1001/jamanetworkopen.2024.43975

**Published:** 2024-10-28

**Authors:** Brock E. Polnaszek, Laurie B. Griffin, Siraj Amanullah, Methodius G. Tuuli, Adam K. Lewkowitz, Annie Gjelsvik

**Affiliations:** 1 Division of Maternal Fetal Medicine, Department of Obstetrics and Gynecology, Medical College of Wisconsin, Milwaukee; 2 Division of Maternal Fetal Medicine, Department of Obstetrics and Gynecology, Women & Infants Hospital, Warren Alpert Medical School of Brown University, Providence, Rhode Island; 3 Department of Emergency Medicine, Pediatrics, and Health Services, Policy and Practice Rhode Island Hospital, Warren Alpert Medical School of Brown University, Providence; 4 Brown University School of Public Health, Providence, Rhode Island

## Abstract

This cross-sectional study investigates the association between parental health and flourishing among children aged 6 months to 5 years in the US.

## Introduction

Flourishing is a key factor for child health trajectory.^1,2,3,4,5^ The National Survey of Children’s Health (NSCH) began collecting measures of flourishing in 2011 and reported 1 in 2 children in the US were flourishing.^1,2^ Subsequently, deciphering factors that promote flourishing became an important public health focus.^1,2,3,4,5,6^ Parental health and childhood flourishing are interrelated, yet elucidating parental health factors that are associated with childhood flourishing is needed.^1,2,3,4,5,6^ Our objective was to examine the association between parental health and flourishing among young children in the US.

## Methods

This cross-sectional study was exempt from review because it used publicly available, deidentified data, in accordance with 45 CFR §46. Nationally representative data were analyzed from 2021 NSCH of young children aged 6 months to 5 years (n = 30 912). Survey respondents provided voluntary consent. We followed the STROBE reporting guideline. The primary outcome was flourishing defined by responding “always/usually” to the following questions: (1) Is this child affectionate and tender? (2) Does this child bounce back quickly when things do not go their way? (3) Does this child show interest and curiosity in learning new things? (4) Does this child smile and laugh? The primary outcome was dichotomized into flourishing (score = 4) and not flourishing (scores 0-3).^6^

The main exposure of composite overall parental health was examined from the father and/or mother response options that included “absent,” “fair/poor,” “good,” or “excellent/very good” to the following questions: “If this child’s mother/father is a primary caregiver and lives in the household, what is the mother’s/father’s physical health status?” The same structure was used to ask about father and/or mother parental mental health status. The responses to the aforementioned 16 questions for each parent were scored with −1 for “absent” to a maximum score of 2 for “excellent/very good” and combined into a composite overall parental health category. Composite scores were then split into tertiles of low (scores −4 to 2), medium (scores 3 to 7), and high (scores of 8). Additional characteristics from previous flourishing literature were assessed including child age, sex, race and ethnicity, adverse experiences, and insurance; and parent and/or caregiver education level, aggravation, emotional support, and coping with daily demands of raising a child; and family household income, family resilience, and smoking in household.^1,2,3,4,5,6^ Weighted baseline composite overall parent health category and flourishing characteristics were compared using the Pearson χ^2^ test for independence. A weighted multivariable logistic regression analysis calculated unadjusted odds ratio (OR) and adjusted OR (aOR) of flourishing by composite overall parental health category. Weighted subgroup analysis explored the association of father’s and mother’s physical and mental health individual responses with odds of flourishing. Several models were run in a stepwise fashion using established variables from prior flourishing literature and eliminated collinearity.^1,2,3,4,5,6^ Two-sided *P* < .05 was considered statistically significant. Statistical analysis was performed using Stata/SE 18 (StataCorp).

## Results

Among 30 912 children aged 6 months to 5 years included in the study, 15 977 (51.7%) were male and 14 935 (48.3%) were female. The majority (81%) of children aged 6 months to 5 years in the US were flourishing in this study. As overall composite parental health category increased, rates of flourishing increased (low: 5454 [75.0%]; medium: 5717 [77.0%]; high: 14 571 [86.0%]; *P* < .001). After adjusting for differences in Table 1 including caregiver level of education, family resilience, parental aggravation, parental coping, and emotional support with parenthood, there were no associations between composite overall parental health category and childhood flourishing. Subgroup analysis found worse mother’s physical health status was associated with reduced flourishing (“poor/very poor”: aOR, 0.68 [95% CI, 0.49-0.95]; “good”: aOR, 0.81 [95% CI, 0.67-0.97]) as compared with responses of “excellent/very good” (Table 2). Father’s physical health of “poor/very poor” was also negatively associated with flourishing (aOR, 0.59 [95% CI, 0.40-0.87]).

**Table 1.** Weighted Parent-Child Characteristics Among Children Aged 6 Months to 5 Years in the US by Composite Overall Parental Health Category^a^

Characteristics	No. (%)			*P* value
	Composite overall parents health score			
	Low (n = 7164)	Medium (n = 7169)	High (n = 16 579)	
Flourishing				
Scores 0-3 (not flourishing)	1710 (25.0)	1452 (23.0)	2008 (35.0)	<.001
Score 4 (flourishing)	5454 (75.0)	5717 (77.0)	14 571 (86.0)	
Child’s age, y				
0	327 (7.6)	362 (7.6)	970 (9.1)	.21
1	804 (17.0)	957 (17.0)	2362 (19.0)	
2	1416 (18.0)	1537 (17.0)	3636 (19.0)	
3	1463 (19.0)	1436 (21.0)	3164 (18.0)	
4	1505 (20.0)	1461 (20.0)	3262 (19.0)	
5	1649 (19.0)	1416 (18.0)	3185 (17.0)	
Child’s sex				
Male	3672 (50.0)	3680 (53.0)	8625 (51.0)	.48
Female	3492 (50.0)	3489 (47.0)	7954 (49.0)	
Child’s race and ethnicity^b^				
Black, non-Hispanic	1077 (25.0)	200 (7.9)	515 (6.9)	<.001
Hispanic	1322 (30.0)	925 (24.0)	1882 (21.0)	
Multiracial, non-Hispanic	1251 (13.0)	919 (11.0)	2214 (12.0)	
White, non-Hispanic	3514 (32.0)	5125 (57.0)	11 968 (60.0)	
Income level of child’s household, % FPL				
0-199	3731 (63.0)	1761 (34.0)	3071 (25.0)	<.001
200-299	1174 (16.0)	1268 (21.0)	2446 (15.0)	
300-399	769 (7.8)	1249 (14.0)	2575 (14.0)	
≥400	1490 (14.0)	2891 (31.0)	8587 (46.0)	
Adequacy of child’s insurance				
Adequate	5285 (74.0)	4809 (68.0)	12 226 (73.0)	<.001
Not adequate	1420 (17.0)	2149 (28.0)	3766 (22.0)	
Uninsured	422 (8.3)	206 (3.7)	562 (5.4)	
Current health insurance status of child				
Insured	6717 (91.0)	6959 (96.0)	16 001 (95.0)	<.001
Not insured	422 (8.3)	206 (3.7)	562 (5.4)	
Consistency of insurance coverage of child				
Consistently insured	6561 (89.0)	6827 (94.0)	15 797 (93.0)	<.001
Currently uninsured/had gap	560 (11.0)	302 (6.2)	649 (6.4)	
Type of health insurance				
Public	3371 (55.0)	1342 (26.0)	2064 (19.0)	<.001
Private	2802 (29.0)	5255 (64.0)	13,374 (72.0)	
Public and private	439 (5.3)	304 (5.6)	455 (3.0)	
Uninsured	422 (8.3)	206 (3.7)	562 (5.4)	
Child has special health care needs				
None	6002 (87.0)	6095 (86.0)	15 051 (92.0)	<.001
Yes, less complex	244 (2.7)	262 (3.1)	462 (1.8)	
Yes, more complex	918 (11.0)	811 (11.0)	1066 (6.4)	
No. of adverse childhood experiences for child				
2 or more	1642 (23.0)	459 (8.0)	232 (1.8)	<.001
1	1959 (26.0)	1258 (21)	1103 (8.4)	
0	3173 (46.0)	5,450 (71.0)	15 237 (90.0)	
Highest education of child’s caregiver				
Less than high school	373 (16.0)	79 (6.4)	187 (3.9)	<.001
High school degree or GED	1733 (30.0)	735 (16.0)	1113 (11.0)	
Some college/technical	2111 (25.0)	1511 (20.0)	2423 (15.0)	
College degree or higher	2947 (29.0)	4844 (57.0)	12 836 (70.0)	
Family resilience index score^c^				
0 or 1	554 (8.3)	395 (7.0)	149 (1.0)	<.001
2 or 3	726 (12.0)	943 (14)	687 (4.2)	
4	5,120 (70.0)	5814 (78.0)	15 681 (94.0)	
Parent coping				
Not very well or not at all	183 (2.3)	209 (3.9)	85 (0.6)	<.001
Somewhat well	2726 (34.0)	3946 (54.0)	5337 (31.0)	
Very well	4,013 (59.0)	3005 (42.0)	11 129 (68.0)	
Parental aggravation				
Usually/always feel aggravation	379 (4.5)	437 (6.7)	453 (2.3)	<.001
Emotional help parenthood				
Yes, spouse/partner	3241 (41.0)	5821 (77.0)	14,396 (83.0)	<.001
Yes, not from spouse/partner	2047 (26.0)	262 (3.5)	269 (1.9)	
Did not receive support	1466 (27.0)	1048 (19.0)	1813 (14.0)	
Coping with daily demands of raising children				
Very well	4013 (59.0)	3005 (42.0)	11 129 (68.0)	<.001
Somewhat well	2726 (34.0)	3946 (54.0)	5337 (31.0)	
Not very well	183 (2.3)	209 (3.9)	5337 (31.0)	
Someone living in household smokes				
Yes	1320 (20.0)	981 (14.0)	1048 (7.6)	<.001
No	5409 (80.0)	6155 (86.0)	15 475 (92.0)	

Abbreviations: FPL, federal poverty level; GED, General Educational Development.

^a^ Children who were institutionalized were not included.

^b^ Self-reported race.

^c^ Family resilience index score based on responses to the following 4 survey items: “When your family faces problems, how often are you likely to do each of the following?” (1) Talk together about what to do, (2) Work together to solve our problems, (3) Know we have strengths to draw on, and (4) Stay hopeful even in difficult times. Response options to the 4 items are: none of the time, some of the time, most of the time, or all of the time. To meet each individual indicator, a response of either most of the time or all of the time was required.

**Table 2.** Weighted Flourishing Among Young Children Aged 6 Months to 5 Years in the United States by Composite Overall and Individual Physical and Mental Parental Health

Parental health status	Not flourishing, index score 0-3	Flourishing, index score 4	Unadjusted odds ratio (95% CI)	Adjusted odds ratio (95% CI)^a^
**Composite overall parental health**				
High	2008 (35.0)	14 571 (52.0)	1 [Reference]	1 [Reference]
Medium	1452 (25.0)	5717 (20.0)	0.55 (0.46-0.65)	0.80 (0.67-0.96)
Low	1710 (40.0)	5454 (28.0)	0.48 (0.41-0.57)	0.83 (0.67-1.03)
**Mother physical health**				
Excellent/very good	2882 (53.0)	18 247 (68.0)	1 [Reference]	1 [Reference]
Good	1297 (23.0)	4542 (17.0)	0.58 (0.59-0.68)	0.81 (0.67-0.97)
Poor/very poor	557 (13.0)	1366 (6.9)	0.42 (0.33-0.55)	0.68 (0.49-0.95)
Absent mother	434 (11.0)	1587 (8.3)	0.56 (0.43-0.74)	0.95 (0.69-1.30)
**Mother mental health**				
Excellent/very good	2526 (50.0)	16 962 (66.0)	1 [Reference]	1 [Reference]
Good	1430 (25.0)	5237 (17.0)	0.54 (0.45-0.64)	0.70 (0.58-0.84)
Poor/very poor	780 (14.0)	1956 (8.8)	0.49 (0.39-0.61)	0.93 (0.71-1.23)
Absent mother	434 (11.0)	1587 (8.3)	0.55 (0.42-0.72)	0.94 (0.68-1.29)
**Father physical health**				
Excellent/very good	2574 (45.0)	16 584 (59.0)	1 [Reference]	1 [Reference]
Good	1053 (18.0)	4359 (16.0)	0.66 (0.55-0.80)	0.86 (0.70-1.06)
Poor/very poor	426 (8.9)	1226 (5.4)	0.46 (0.35-0.62)	0.59 (0.40-0.87)
Absent father	1117 (28.0)	3573 (20.0)	0.54 (0.45-0.65)	0.84 (0.66-1.07)
**Father mental health**				
Excellent/very good	2618 (49.0)	16 875 (62.0)	1 [Reference]	1 [Reference]
Good	949 (15.0)	3834 (13.0)	0.67 (0.55-0.81)	0.88 (0.72-1.07)
Poor/very poor	486 (8.1)	1460 (5.9)	0.58 (0.43-0.77)	0.86 (0.58-1.29)
Absent father	1117 (28.0)	3573 (20.0)	0.56 (0.47-0.68)	0.87 (0.68-1.11)

^a^ Adjusted for caregiver education, family resilience, parental coping, parental aggravation, and emotional support.

## Discussion

Mother’s reported physical health status was associated with flourishing among young children in the US. Further investigation into the interplay of parent health, family unit, and social determinants of health such as education, income, and caregiver support are warranted. Limitations include single period, caregiver reported, and exclusion of children in long-term facilities.

## Notes

Supplement.
Data Sharing Statement
