# Supplementary material for: Parental Health and Flourishing Among Young US Children
Source: JAMA Netw Open. 2024 Oct 28;7(10):e2443975. doi: 10.1001/jamanetworkopen.2024.43975 (PMC11581517; doi:10.1001/jamanetworkopen.2024.43975)
Supplement: Supplement. — Data Sharing Statement [file jamanetwopen-e2443975-s001.pdf]

Data Sharing Statement

Polnaszek. Parental Health and Flourishing Among Young US Children. *JAMA Netw Open*. Published October 28, 2024. doi:10.1001/jamanetworkopen.2024.43975

Data
Data available: Yes
Data types: Data dictionary
How to access data: Available from NSCH website
When available: With publication

Supporting Documents
Document types: Statistical/analytic code
How to access documents: Bpolnaszek@mcw.edu
When available: With publication

Additional Information
Who can access the data: Anyone requesting
Types of analyses: Any purpose
Mechanisms of data availability: With investigator support
